# Publication statuses of clinical trials supporting FDA-approved immune checkpoint inhibitors: a meta-epidemiological investigation

**DOI:** 10.1186/s12885-019-6232-x

**Published:** 2019-10-24

**Authors:** Kenji Omae, Yuki Kataoka, Yasushi Tsujimoto, Yusuke Tsutsumi, Yosuke Yamamoto, Shunichi Fukuhara, Toshi A. Furukawa

**Affiliations:** 1 Department of Innovative Research and Education for Clinicians and Trainees (DiRECT), Fukushima Medical University Hospital, 1 Hikarigaoka, Fukushima city, Fukushima, 960-1295 Japan; 2 Department of Healthcare Epidemiology, Kyoto University School of Public Health in the Graduate School of Medicine, Kyoto, Japan; 3 Department of Urology, Tokyo Women’s Medical University, Tokyo, Japan; 4 Hospital Care Research Unit, Hyogo Prefectural Amagasaki General Medical Center, Hyogo, Japan; 5 Department of Nephrology and Dialysis, Kyoritsu Hospital, Hyogo, Japan; 6 Department of Emergency Medicine, National Hospital Organization Mito Medical Center, Ibaraki, Japan; 7 Department of Health Promotion and Human Behavior, Kyoto University School of Public Health in the Graduate School of Medicine, Kyoto, Japan

**Keywords:** Anticancer drugs, Clinical trials, Drug approval, Immune checkpoint inhibitors, Publications, United states food and drug administration

## Abstract

**Background:**

The low data publication rate for Food and Drug Administration (FDA)-approved drugs, and discrepancies between FDA-submitted versus published data, remain a concern. We investigated the publication statuses of sponsor-submitted clinical trials supporting recent anticancer drugs approved by the FDA, with a focus on immune checkpoint inhibitors (ICPis).

**Methods:**

We identified all ICPis approved between 2011 and 2014, thereby obtaining 3 years of follow-up data. We assessed the clinical trials performed for each drug indication and matched each trial with publications in the literature. The primary benchmark was the publication status 2 years post-approval. We examined the association between time to publication and drug type using a multilevel Cox regression model that was adjusted for clustering within drug indications and individual covariates.

**Results:**

Between 2011 and 2014, 36 anticancer drugs including 3 ICPis were newly approved by the FDA. Of 19 trials investigating the 3 ICPis, 11 (58%) were published within 2 years post-approval. We randomly selected 10 of the 33 remaining anticancer drugs; 68 of 101 trials investigating these drugs (67%) were published. Overall, the publication rate was 66% at 2 years post-approval with a median time to publication of 2.3 years. There was no significant difference in the time to trial publication between ICPis and other anticancer drugs (adjusted hazard ratio [HR], 1.1; 95% confidence interval [CI], 0.8–1.7; *P* = 0.55). However, findings related to non-ICPis investigated specifically in randomized phase 2 or phase 3 trials were significantly more likely to be published earlier than those related to ICPis (adjusted HR, 7.4; 95% CI, 1.8–29.5; *P* = 0.005).

**Conclusion:**

One in 3 sponsor-submitted trials of the most recently approved anticancer drugs remained unpublished 2 years post-FDA approval. We found no evidence that the drug type was associated with the time to overall trial publication.

## Background

An improved understanding of the biology of cancer has led to remarkable progress in therapeutic approaches. Anticancer agents developed over the last 2 decades utilize multiple mechanisms of action including conventional cytotoxic agents as well as inhibition of oncogenic signalling pathways and angiogenesis. More recently, ‘immunotherapy’ agents that rely on immunomodulatory mechanisms to target and destroy cancer cells, most notably immune checkpoint inhibitors (ICPis), have been developed.

The first ICPi approved by the United States Food and Drug Administration (FDA) was ipilimumab, a fully humanized immunoglobulin G1 monoclonal antibody that blocks cytotoxic T-lymphocyte antigen [1]. Pembrolizumab and nivolumab were the first ICPis that target programmed cell death protein 1; they showed high response rates with favourable toxicity profiles and were approved for treating metastatic melanoma in 2014 [2, 3]. The notable successes of these pivotal trials may have led to unrealistically high expectations among patients and clinicians, as more recent studies have shown that only a subset of patients exhibit durable responses, and existing checkpoint-blocking monotherapies seldom lead to complete remission [4–6]. These findings have prompted the search for next-generation ICPis as well as evaluations of their combinations with other biologic agents [7].

Anticancer drugs are approved by the FDA based on substantial evidence of clinical benefit from adequate and well-controlled clinical trials. Their efficacies are demonstrated by prolonging patients’ survival and improving their quality of life by preventing or ameliorating cancer-related symptoms. Sponsors of a new drug are required to submit all data to the FDA, including complete protocols, protocol revisions, and data from successful and failed trials. Once the drug is approved, the FDA produces a ‘Summary Basis of Approval’ document that contains synopses and evaluations of clinical data and statistical analyses performed by FDA medical officers during the approval process. These documents contain detailed efficacy and safety data that are relevant to drug approval but are not necessarily intended to be shared with general evidence users such as clinicians, patients, and policymakers. In this context, the peer-reviewed medical literature has a powerful and important role in disseminating information relevant to both clinicians and the public. Nevertheless, the publication rates of sponsor-submitted trial results for drugs approved by the FDA have been low, and discrepancies exist between original trial data submitted to the FDA and data found in published trials [8–10]. The lack of timely and complete dissemination of clinical trial data can lead to unnecessary duplication of research and impair evidence-based clinical decision-making, thus violating ethical obligations. Delayed and incomplete dissemination can have particularly deleterious effects on cancer patients.

Thus, we performed a comprehensive examination of the publication statuses of trials submitted by the sponsors of investigating the most recent FDA-approved anticancer drugs, with a focus on ICPis. As we hypothesized that the growing enthusiasm around ICPis may lead to expediting the publication of data involving these drugs, we further evaluated the role of the drug types in the time taken to publish their associated clinical trial results.

## Methods

The protocol for this meta-epidemiological investigation was registered with the University Hospital Medical Information Network (www.umin.ac.jp/ctr/index-j.htm; registration number UMIN000030475).

### Drug analysis

We used the Drugs@FDA database to identify all ICPis that were newly approved for cancer treatment by the FDA between 2011 (the year the first ICPi was approved by the FDA) and 2014 (thus assuring a follow-up of at least 3 years post-approval). All other anticancer drugs approved by the FDA between 2011 and 2014 were also identified, 10 of which were randomly selected for comparison using the Excel software (Microsoft Corp, Redmond, WA, USA). We included only new drugs against novel molecular targets and excluded those that are preventative or palliative.

### Identification of clinical trials

We retrieved the FDA Summary Basis for Approval of each drug and assessed medical review documents to identify clinical trials submitted by the sponsor. The medical reviews included an overview of safety and efficacy, an outline of the data sources, integrated summaries of safety and efficacy, and (where relevant) a description of individual clinical trials. We included trials that were or were not covered by the Food and Drug Administration Amendments Act of 2007 (FDAAA) mandate for submission of results (efficacy trials: phase 2–3) [11], because the nonpublication of any clinical trial stage has potentially deleterious impacts on patients and clinicians, represents a waste of resources, and violates ethical imperatives to share results. Ethical board review and informed consent were not required for this survey of publicly available databases and articles in which aggregated data were inherently anonymized.

### Search strategy and data extraction

First, we recorded the following characteristics for each submitted trial when available in FDA documents: the drug name (generic and trade), initial approval date, approval characteristics (FDA review process and approval pathway), drug target, delivery method, dosage and evaluation schedules, indication, number and location of study sites, sponsors’ and principal investigators’ names, authors’ industry affiliations, study phase, study type (superiority, non-inferiority, or equivalence trial), number of arms, control conditions, number of study participants, primary and secondary outcomes, sample size in the primary analysis, and effect size of each primary outcome. Second, using the above information as search terms, we electronically searched PubMed, Google/Google Scholar, and their sponsors’ websites to obtain study identifiers (ClinicalTrials.gov registry [NCT] number and/or trial unique ID) for each trial identified in the FDA review documents.

Next, we searched ClinicalTrials.gov and the World Health Organization International Clinical Trials Registry Platform with the study identifier to obtain the following detailed information for each trial: dosing schedules, number and location of study centres, principal investigators’ names, authors’ industry affiliations, study phase, study type (superiority, non-inferiority, or equivalence trial), number of arms, control conditions, planned sample sizes, compared parameters, number of study participants, primary and secondary outcomes, sample size in the primary analysis, effect size of the primary outcome, statistical significance of the primary outcome (*P* < 0.05 or confidence interval [CI] excluding those with ‘no difference’; or if the study was a non-inferiority evaluation, the CI including ‘no difference’ and excluding the prespecified margin described in the protocol; or if the study was an equivalence evaluation, the CI between the no difference and prespecified margin). Nonsignificant or null results were defined as *P* > 0.05 or a CI including ‘no difference’, or else a CI including the prespecified margin if the study investigated non-inferiority or its equivalent. We also noted whether the trial was randomized and/or double-blinded. Missing, unclear, or important additional data were requested from sponsors or primary study authors.

### Publication matching

We searched PubMed, Google/Google Scholar, and their sponsors’ websites to match each identified trial to publications in the medical literature between June and August 2018. We also searched abstracts in the proceedings of relevant periodic meetings as well as reference lists. Studies in all languages were reviewed as abstracts or full texts. Trials identified in FDA documents were matched to publications based on the following characteristics: study identifier (NCT number and/or trial ID), drug name, sample size, dosing schedules, arm number, primary and secondary outcome measures, and statistical significance or estimated effect of the primary outcome results. The publication type of each trial was recorded as follows: (1) full publication, (2) full report, (3) partial publication, (4) conference abstract, (5) none (neither published nor reported, but verified), or (6) unclear (no information found). Only original research reports in full peer-reviewed journals were considered full publications and included all the primary outcomes predefined in the protocol (#1 above) or partial publications containing incomplete descriptions of the prespecified primary outcomes (#3 above). For trials that were terminated early because of perceived effectiveness, only original research reports were considered full publications (#1 above) including all findings and results. If all the predefined primary outcomes were available in ClinicalTrials.gov or the sponsors’ websites, the trial was considered a full report (#2 above). If multiple publications were found for the same trial, we prioritized the category with the smaller number; for example, if a trial was fully reported (#2 above) and published (#1 above), then it was categorized as a full publication (#1 above). If trials remained unmatched to a publication, we contacted the sponsors or authors to clarify their publication statuses. Four reviewers (KO, YK, YT, and YT) screened all abstracts and full-text articles independently. Disagreements were resolved by discussion; otherwise, a fifth independent reviewer (TAF) arbitrated.

### Statistical analysis

We performed descriptive statistics of the included trials stratified by drug type (ICPis vs. other anticancer drugs). The primary endpoint was the rate of ‘full publication’ within 2 years after FDA approval [9]; we also analysed the publication statuses at 0 and 3 years. Moreover, we evaluated whether study identifiers were reported to determine the articles’ discoverability; for example, once a trial’s NCT number is published as part of the original journal article, it is automatically identified and indexed by ClinicalTrials.gov.

Next, we examined the influence of study phase and drug type on the time from FDA approval to ‘full publication’ using log-rank tests. In time-to-event analyses, trials that were not published were censored, and time 0 was defined as the date of FDA approval per the Administration’s documents. Trials published before their FDA approval date were considered published at time 0.

We further performed multivariable analysis of the association between drug type/study phase and time to publication using a multilevel Cox regression model that was adjusted for clustering within drug indications and potential confounders, including sample size and ethnicity. We classified trials as ‘smaller’ if the sample size was smaller than the median value of all the studies combined; otherwise, they were deemed ‘larger’.

We conducted a limited number of prespecified subgroup and sensitivity analyses and examined the time to publication among all as well as randomized phase 2/3 trials. The sensitivity analyses employed a multilevel ordered logistic regression model to evaluate the association between drug type and publication status according to the abovementioned categories (categories 5 and 6 were combined) at 0, 2, and 3 years with adjustment for clustering within drug indications and individual covariates. Additionally, we performed a post-hoc analysis of the “full publication” rate at 2 years post-approval of trials that supported only the drug indications for which priority review was granted by the FDA; this was to determine the impact of such priority review on the time to publication. Statistical significance was set at *P* < 0.05 (2-tailed test). We used STATA version 14 (Stata Corp LP, College Station, TX, USA) for our analyses.

## Results

### Sample characteristics

**Fig. 1:**
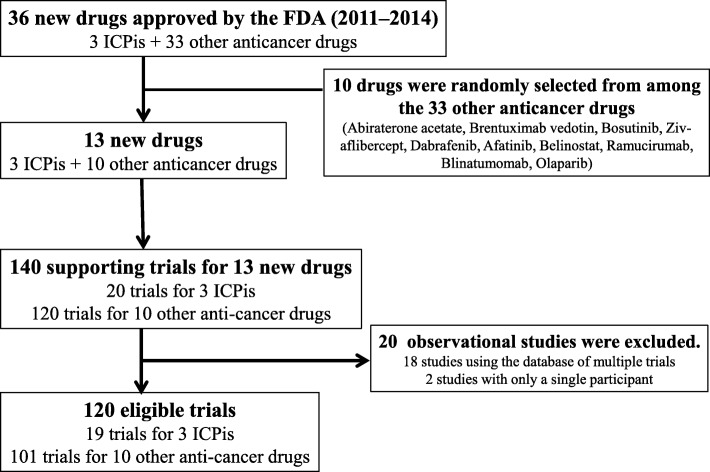
Flowchart showing the selection of new drugs and supporting trials ICPi, immune checkpoint inhibitor

The FDA approved 3 ICPis and 33 other anticancer drugs between 2011 and 2014; 10 of the latter were randomly selected for this study. We identified 140 trials in the FDA review documents supporting their drug approval; 120 trials (19 for ICPis and 101 for other anticancer drugs) were ultimately eligible for this study (Fig. 1). Table 1 summarizes the characteristics of the included drugs and their supporting trials as submitted by the sponsor. All 3 ICPis (100%) received orphan drug status; 2 (67%) were breakthrough therapies and 2 (67%) received accelerated approval. Among the 10 non-ICPis, orphan drug and breakthrough therapy statuses were granted to 7 (70%) and 1 (10%), respectively, while priority review and accelerated approval were granted to 4 drugs each (40%). ICPi trials were more likely to be late-phase, randomized, and double-blinded studies with larger cohorts. Nearly all trials reported adverse events, and a majority had authors affiliated with the pharmaceutical industry. Over 20% did not report all predefined outcomes (i.e. engaged in selective outcome reporting).

**Table 1:** Characteristics of included trials by anticancer drug type

	ICPi (*n* = 3)	Other anticancer drug (*n* = 10)
Included drugs (n)	Ipilimumab, Pembrolizumab, Nivolumab	Abiraterone acetate, Brentuximab vedotin, Bosutinib, Ziv-aflibercept, Dabrafenib, Afatinib, Belinostat, Ramucirumab, Blinatumomab, Olaparib
Drug approval characteristics, n (%)	Priority review 0, orphan drug 3 (100%), breakthrough therapy 2 (67%), accelerated approval 2 (67%)	Priority review 4 (40%), orphan drug 7 (70%), breakthrough therapy 1 (10%), accelerated approval 4 (40%)
	ICPi trials (*n* = 19)	Other anticancer drug trials (*n* = 101)
Study phase, n (%)		
Phase 1	5 (26)	42 (42)
Phase 2	9 (47)	48 (48)
Phase 3	5 (26)	11 (11)
Efficacy study, n (%)	11 (58)	18 (18)
Randomized study, n (%)	12 (63)	28 (28)
Double-blinded study, n (%)	7 (37)	13 (13)
Multi-country study, n (%)	12 (63)	52 (51)
Author affiliation with industry, n (%)	18 (95)	82 (81)
Sample size, median (IQR)	284 (127–676)	58 (37–121)
Statistically significant outcomeª, n (%)	8 (42)	10 (10)
Reporting of adverse events, n (%)	19 (100)	91 (90)
Selective outcome reporting, n (%)	6 (32)	21 (21)

*ICPi* immune checkpoint inhibitor, *IQR* interquartile range

ªAt least 1 of the primary outcomes was statistically significant

### Study identifiers

Eighteen of 89 published trials (20%) lacked a study identifier (Table 2). All phase 3 trial articles and those reporting a statistically significant primary outcome included an NCT number and/or trial ID. Notably, all articles on ICPi trials except 1 also described the study identifier; however, 24% of the articles on anticancer drug trials had no such identifiers.

**Table 2:** Characteristics of fully published trials according to whether the study identifier is present

	Presence or absence of study identifier in the published article	
	Yes (*n* = 71)	No (*n* = 18)
Study phase, n (%)		
Phase 1	17 (24)	12 (67)
Phase 2	38 (54)	6 (33)
Phase 3	16 (23)	0
Drug type, n (%)		
Immune checkpoint inhibitors	18 (25)	1 (6)
Other anticancer drugs	53 (75)	17 (94)
Sample size, median (interquartile range)	102 (53–345)	42 (37–56)
Multi-country study, n (%)	46 (65)	5 (28)
Author affiliation with industry, n (%)	71 (100)	16 (89)
Statistically significant outcomeª, n (%)	17 (24)	0
Reporting of adverse events, n (%)	71 (100)	18 (100)
Selective outcome reporting, n (%)	8 (11)	7 (39)

ªAt least 1 of the primary outcomes was statistically significant

### Publication status

Table 3 shows the publication status at 0, 2, and 3 years post-FDA approval. Overall, 41 trials (34%) had not been published in full by 2 years post-approval; over 40% of ICPi trials remained unpublished. We categorized 2 trials for other anticancer drugs as unclear because, although we identified publications describing their results, the trials themselves had not been documented in any registry and no protocol was available. Therefore, we were unable to identify their primary outcomes and could not determine their publication status according to our classification.

**Table 3:** Publication status of included trials at 0, 2, and 3 years post-approval

	0 years			2 years			3 years		
Publication status, n (%)	ICPi trials	Other anticancer drug trials	All trials	ICPi trials	Other anticancer drug trials	All trials	ICPi trials	Other anticancer drug trials	All trials
Full publication	6 (32)	35 (35)	41 (34)	11 (58)	68 (67)	79 (66)	15 (79)	71 (70)	86 (72)
Full report	0	10 (10)	10 (8)	2 (11)	12 (12)	14 (12)	2 (11)	11 (11)	13 (11)
Partial publication	5 (26)	5 (5)	10 (8)	5 (26)	6 (6)	11 (9)	2 (11)	7 (7)	9 (8)
Conference abstract	2 (11)	14 (14)	16 (13)	0	3 (3)	3 (3)	0	2 (2)	2 (2)
None	6 (32)	35 (35)	41 (34)	1 (5)	10 (10)	11 (9)	0	8 (8)	8 (7)
Unclear	0	2 (2)	2 (2)	0	2 (2)	2 (2)	0	2 (2)	2 (2)

*ICPi* immune checkpoint inhibitor

### Trial characteristics associated with time to publication

**Fig. 2:**
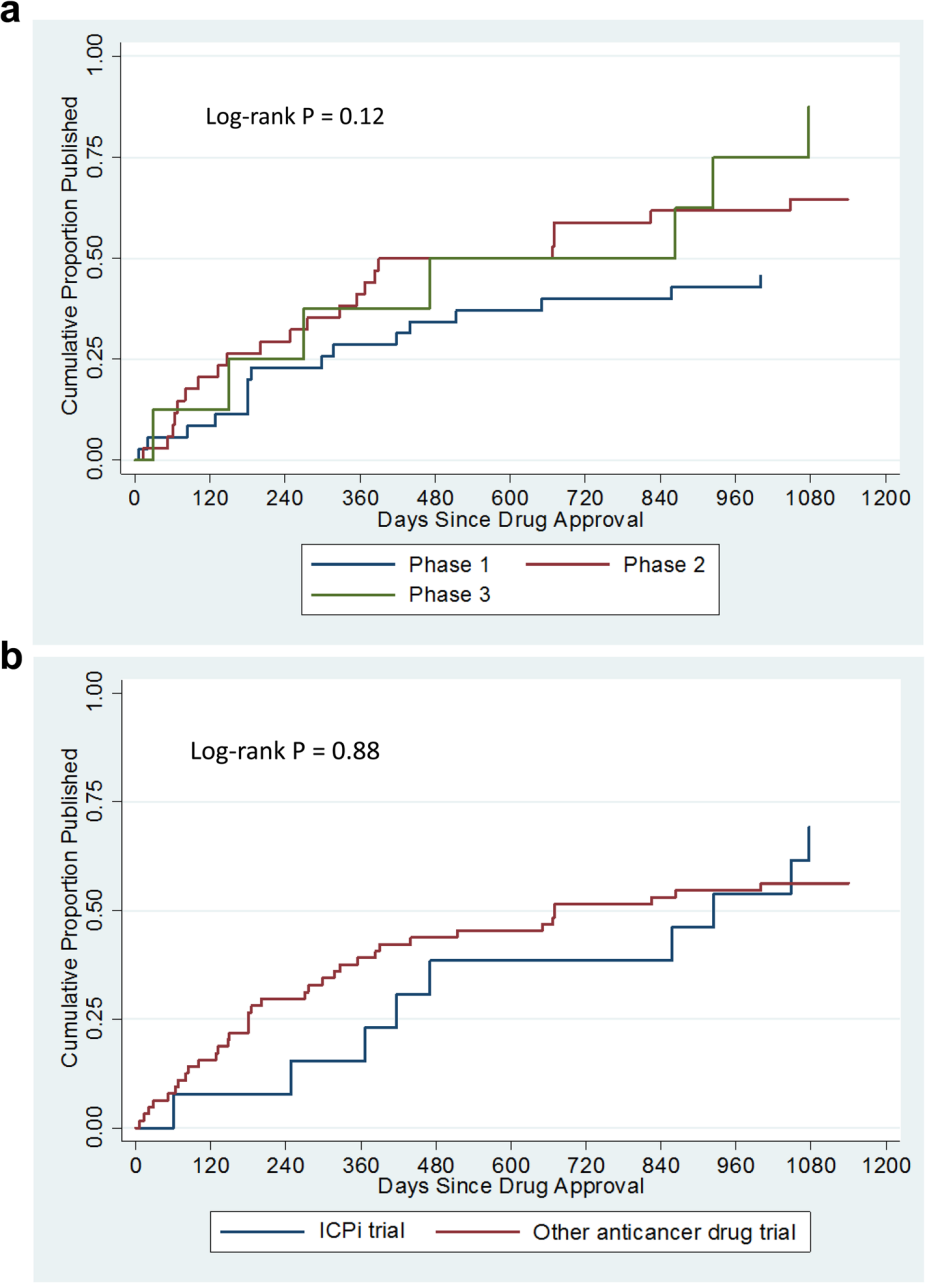
Daily publications of trials supporting the approval of new anticancer drugs (**a**) Daily publications by study phase. (**b**) Daily publications by drug type. ICPi, immune checkpoint inhibitor

The median time from FDA approval to ‘full publication’ was 2.3 years (interquartile range, 6.7 months to not estimable). Figure 2 shows the cumulative proportion of fully published trials by phase and drug type. Neither the trial phase nor the drug type significantly affected the time to publication.

A multivariable Cox regression model analysis confirmed no significant difference in the time to trial publication between ICPis and other anticancer drugs (adjusted hazard ratio [HR] of other anticancer drugs, 1.1; *P* = 0.55). However, when controlled for confounders, phase 2 or 3 trials were published faster than phase 1 trials (adjusted HR, 1.7; *P* = 0.02) (Table 4).

**Table 4:** Characteristics associated with full publication: Cox proportional hazards model analysis

	HR (95% CI)	*P*-value
Drug type		
ICPi	ref.	
Other anticancer drugs	1.1 (0.8–1.7)	0.55
Study phase		
Phase 1	ref.	
Phase 2 or 3	1.7 (1.1–2.6)	0.02
Multi-country study		
No	ref.	
Yes	1 (0.4–2.2)	0.95
Sample size		
Smaller	ref.	
Larger	1.4 (0.8–2.2)	0.16

*ICPi* immune checkpoint inhibitor, *HR* hazard ratio, *CI* confidence interval, *ref.* reference

### Subgroup analyses

**Fig. 3:**
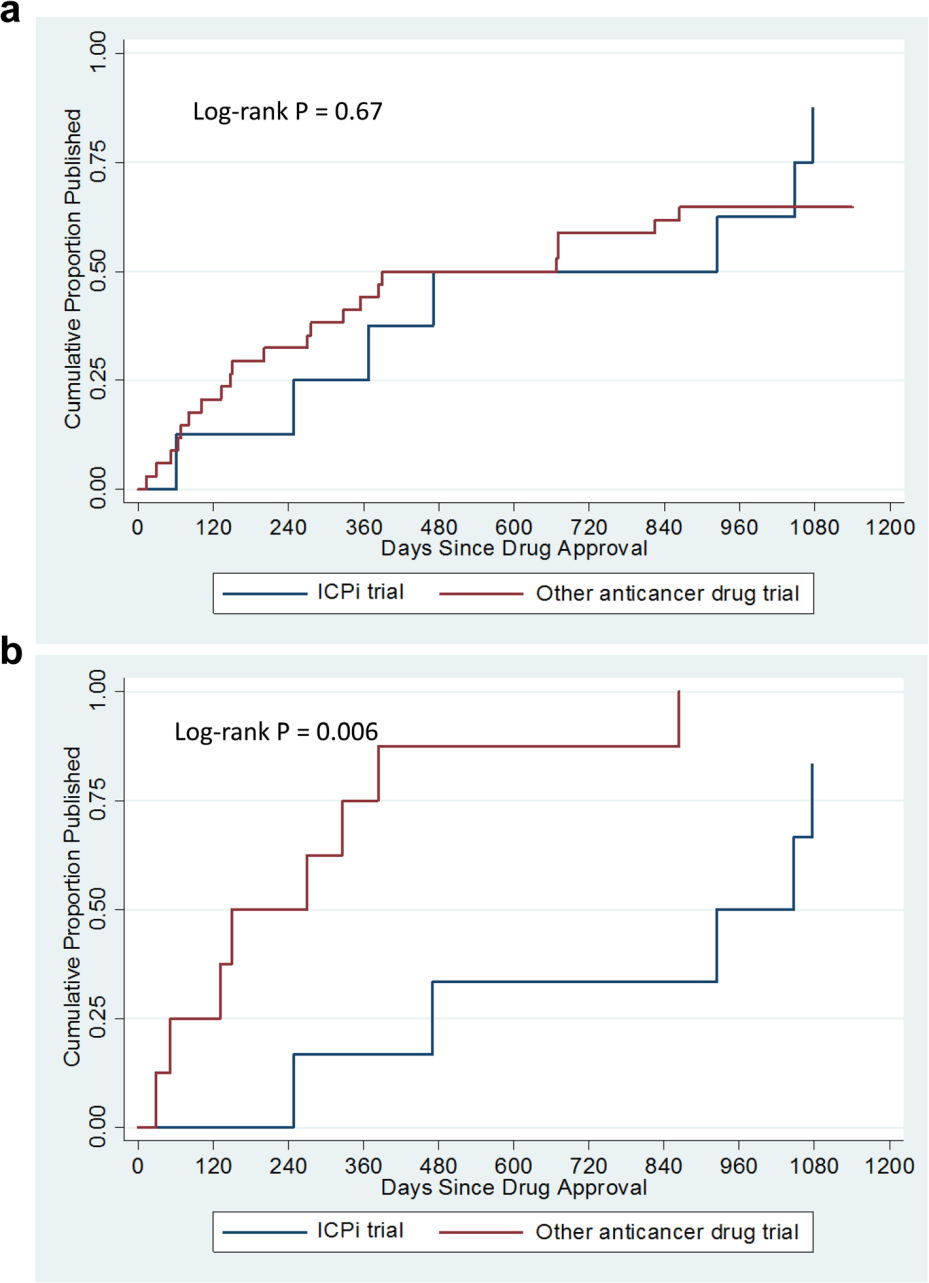
Daily publications of phase 2 and 3 trials supporting the approval of new anticancer drugs (**a**) Daily publications of all phase 2 and 3 trials by drug type. (**b**) Daily publications of randomized-only phase 2 and 3 trials by drug type. ICPi, immune checkpoint inhibitor

Figure 3 shows the cumulative proportion of full publications among all and randomized-only phase 2/3 trails. Randomized phase 2 and 3 trials of other anticancer drugs were published significantly earlier than ICPi trials (*P* = 0.006).

### Sensitivity analyses

Sensitivity analyses confirmed that drug type was not associated with the ordered publication status at 0, 2, or 3 years post-approval (adjusted odds ratio [OR] of other anticancer drugs, 1.1, 1.4, and 0.6 [*P* = 0.92, 0.58, and 0.49], respectively). However, the study phase was significantly associated with the ordered publication status at 2 and 3 years (adjusted OR of phase 2 or 3 trials, 3.1 and 4.6 [*P* = 0.04 and 0.01], respectively); these data are supplied in an additional table [See Additional file 1]. Although we found no association between the drug type and time to publication of phase 2 and 3 trials (adjusted HR, 1.1, *P* = 0.95), other anticancer drugs were associated with significantly earlier publication of randomized phase 2 and 3 trials (adjusted HR, 17.7, *P* < 0.0001); these data are supplied in additional tables [See Additional file 2 and Additional file 3].

### Post-hoc analyses

Of the 46 trials supporting 4 drug indications to which priority review was granted by the FDA, 16 (35%) had not been published in full at 2 years post-approval.

## Discussion

The median time from FDA approval to full publication of the 120 trials supporting the 3 ICPis and 10 randomly selected non-ICPi drugs was 2.3 years, and one-third of the trials remained unpublished 2 years post-approval. Although we found no association between any drug type and time to publication overall, the publication of randomized phase 2 and 3 trials for ICPis took longer than for other anticancer drug types. Interestingly, the publication rates of all trials were very similar, including for those supporting drug indications to which priority review was granted by the FDA.

A previous study found that over half of the trials supporting new drugs approved between 1998 and 2000 remained unpublished ≥5 years after approval, and that statistically significant results were more likely to be reported [9]. Another study found that nearly half of phase 2 and 3 trials for antidepressant agents approved between 1987 and 2004 were unpublished, and possible selective reporting biases were present [12]. Additionally, 97% of clinical trials for cardiovascular disease and diabetes drugs were published in the peer-reviewed literature after the FDAAA was implemented [13].

The publication rate revealed in our investigation was higher than those found in 2 earlier studies performed before the FDAAA implementation [9, 12]. The statistical significance of the results was not associated with earlier trial publication, suggesting an improvement in the dissemination and transparency of trial results related to FDA approval. However, the overall publication rate of 66% remains insufficient to satisfy the responsibilities of medical and academic enterprises. Recent research on all pharmaceutical and biopharmaceutical trials registered with clinicaltrials.gov demonstrated that publication rates varied substantially depending on the disease area, and that oncology-related trials had the lowest publication rates [14]. Stakeholders, including researchers and sponsors as well as journals, ethical committees, and governments, ought to invest additional effort to promote the timely and complete dissemination of clinical trial findings, especially those related to oncology.

Including all the clinical trial that supported drug approval, as required by the Declaration of Helsinki [15], enabled us to quantify the differences in the timing of trial publication across study phases. We also clarified the differences in the discoverability and accessibility of published articles according to study phases. Although previous investigators have described the underreporting of trial registration numbers in biomedical publications related to randomized clinical trials (RCTs) [16, 17], the current study expanded the scope of research to all clinical trials (including RCTs and non-RCTs), and found that such study identifiers were less frequently included in articles describing earlier-phase trials. This suggests that systematically searching for trials (especially earlier ones) using study identifiers is unreliable and could result in undercounting publications and in incomplete data dissemination. Authors and sponsors are encouraged to include study identifiers in all their articles regardless of the study phase or statistical significance of study outcomes.

The results of randomized phase 2 and 3 trials are usually considered ‘gold standard’ evidence of drug efficacy, and thus directly affect both drug marketing approval as well as drug sales. In our study, subgroup analyses of randomized phase 2 and 3 trials showed that the drug type (ICPi vs. non-ICPi) was associated with time to publication; the difference remained significant after adjusting for trial-level confounders. We speculate that the novel ICPi mechanism of action may have influenced each step of the trials’ publication processes, especially as various stakeholders were involved. Recently disclosed details of sponsored trial publication histories indicated that some industry sponsors require the timely submission of all trial results for publication [18, 19]. Evaluators of the dissemination and transparency of clinical trial results should consider such publication-related policies.

Our study had several limitations. First, it was restricted to trials supporting FDA approval of anticancer drugs; therefore, our results are not generalizable. Second, because we focused on recently approved drugs, follow-up times were limited; as such, longer follow-up may yield additional publications (although they may not qualify as timely). Third, our analysis may have been statistically underpowered to detect significant relationships or differences given the limited number of trials. Fourth, it remains possible that we missed some published studies. Lastly, as is inherent in all observational studies, causal inferences cannot be made, and additional unmeasured variables may explain the differences in times to publication.

However, our study also has several strengths, such as the inclusion of all trials irrespective of study phase as well as rigorous search algorithms and thorough statistical analyses.

In conclusion, our results showed that incomplete transparency and delays in disseminating sponsor-submitted clinical trials supporting FDA drug approval are still prevalent. Further efforts and continuous monitoring are necessary to improve the timely and complete publication of clinical trial results.

## Supplementary information

**Additional file 1: Table S1.** Multivariate ordered logistic regression analysis of characteristics associated with trial publication status.

**Additional file 2: Table S2.** Cox proportional hazards model analysis of characteristics of fully published phase 2 and 3 trials.

**Additional file 3: Table S3.** Cox proportional hazards model analysis of fully published randomized phase 2 and 3 trials.

## Data Availability

All analysed data from this study are included in this published article and its additional files. All data generated during the current study are available from the corresponding author on reasonable request.

## Abbreviations

CI: Confidence interval
FDA: Food and Drug Administration
FDAAA: FDA Amendment Act
HR: Hazards ratio
ICPi: Immune checkpoint inhibitor
OR: Odds ratio
RCT: Randomized controlled trial
