# Supplementary material for: Publication statuses of clinical trials supporting FDA-approved immune checkpoint inhibitors: a meta-epidemiological investigation
Source: BMC Cancer. 2019 Oct 24;19:998. doi: 10.1186/s12885-019-6232-x (PMC6814120; doi:10.1186/s12885-019-6232-x)
Supplement: Supplementary file 1 — Additional file 1: Table S1. Multivariate ordered logistic regression analysis of characteristics associated with trial publication status. [file 12885_2019_6232_MOESM1_ESM.doc]

Table S1. Multivariate ordered logistic regression analysis of characteristics associated with trial publication status

|  | 0 years post-approval | | |  | 2 years post-approval | | |  | 3 years post-approval | | |
| --- | --- | --- | --- | --- | --- | --- | --- | --- | --- | --- | --- |
|  | OR (95% CI) | | P-value |  | OR (95% CI) | | P-value |  | OR (95% CI) | | P-value |
| Drug type |  | |  |  |  |  |  |  |  |  |  |
| ICPi |  | ref. |  |  | ref. | |  |  | ref. | |  |
| Other anticancer drugs | 1.1 (0.2–5.5) | | 0.92 |  | 1.4 (0.4–5.3) | | 0.58 |  | 0.6 (0.1–3.0) | | 0.49 |
| Study phase |  |  |  |  |  |  |  |  |  |  |  |
| Phase 1 | ref. | |  |  | ref. | |  |  | ref. | |  |
| Phase 2 or 3 | 2.6 (1.0–7.2) | | 0.06 |  | 3.1 (1.0–9.0) | | 0.04 |  | 4.6 (1.4–14.8) | | 0.01 |
| Multi-country study |  |  |  |  |  |  |  |  |  |  |  |
| No | ref. | |  |  | ref. | |  |  | ref. | |  |
| Yes | 0.9 (0.3–2.5) | | 0.88 |  | 1 (0.3–3.4) | | 0.99 |  | 1.1 (0.3–4.2) | | 0.88 |
| Sample size |  |  |  |  |  |  |  |  |  |  |  |
| Smaller | ref. | |  |  | ref. | |  |  | ref. | |  |
| Larger | 0.6 (0.2–1.5) | | 0.26 |  | 1.1 (0.4–3.0) | | 0.89 |  | 0.9 (0.3–2.9) | | 0.91 |

ICPi, immune checkpoint inhibitor; OR, odds ratio; CI, confidence interval; ref., reference
