# Supplementary material for: Publication statuses of clinical trials supporting FDA-approved immune checkpoint inhibitors: a meta-epidemiological investigation
Source: BMC Cancer. 2019 Oct 24;19:998. doi: 10.1186/s12885-019-6232-x (PMC6814120; doi:10.1186/s12885-019-6232-x)
Supplement: Supplementary file 2 — Additional file 2: Table S2. Cox proportional hazards model analysis of characteristics of fully published phase 2 and 3 trials. [file 12885_2019_6232_MOESM2_ESM.doc]

Table S2. Cox proportional hazards model analysis of characteristics of fully published phase 2 and 3 trials

|  | HR (95% CI) | | P-value |
| --- | --- | --- | --- |
| Drug type |  |  |  |
| ICPi | ref. | |  |
| Other anticancer drugs | 1.1 (0.2–5.2) | | 0.95 |
| Study phase |  |  |  |
| Phase 2 | ref. | |  |
| Phase 3 | 0.8 (0.4–1.7) | | 0.56 |
| Multi-country study |  |  |  |
| No | ref. | |  |
| Yes | 0.9 (0.5–1.6) | | 0.65 |
| Randomized study |  |  |  |
| No | ref. | |  |
| Yes | 3.8 (1.3–11.4) | | 0.02 |
| Sample size |  |  |  |
| Smaller | ref. | |  |
| Larger | 1.2 (0.5–2.9) | | 0.75 |
| Statistically significant outcomeª |  |  |  |
| No | ref. | |  |
| Yes | 0.4 (0.09–2.3) | | 0.33 |

ICPi, immune checkpoint inhibitor; HR, hazard ratio; CI, confidence interval; ref., reference

ªAt least 1 of the primary outcomes was statistically significant.
