# Supplementary material for: Publication statuses of clinical trials supporting FDA-approved immune checkpoint inhibitors: a meta-epidemiological investigation
Source: BMC Cancer. 2019 Oct 24;19:998. doi: 10.1186/s12885-019-6232-x (PMC6814120; doi:10.1186/s12885-019-6232-x)
Supplement: Supplementary file 3 — Additional file 3: Table S3. Cox proportional hazards model analysis of fully published randomized phase 2 and 3 trials. [file 12885_2019_6232_MOESM3_ESM.doc]

Table S3. Cox proportional hazards model analysis of fully published randomized phase 2 and 3 trials

|  | HR (95% CI) | | P-value |
| --- | --- | --- | --- |
| Drug type |  |  |  |
| ICPi | ref. | |  |
| Other anticancer drugs | 17.7 (3.8–82.8) | | <0.0001 |
| FDA approval status |  |  |  |
| Not accelerated | ref. | |  |
| Accelerated | 3.2 (1.5–6.6) | | 0.002 |
| Double-blinded study |  | |  |
| No | ref. | |  |
| Yes | 1.5 (0.6–3.5) | | 0.38 |
| Statistically significant outcomeª |  |  |  |
| No | ref. | |  |
| Yes | 0.9 (0.2–4.7) | | 0.94 |

ICPi, immune checkpoint inhibitor; HR, hazard ratio; CI, confidence interval; ref., reference

ªAt least 1 of the primary outcomes was statistically significant.
